# Targeting resistant breast cancer stem cells in a three-dimensional culture model with oleuropein encapsulated in methacrylated alginate microparticles

**DOI:** 10.1007/s40199-024-00512-3

**Published:** 2024-05-09

**Authors:** Ozlem Altundag-Erdogan, Rumeysa Tutar, Elif Yüce, Betül Çelebi-Saltik

**Affiliations:** 1 Department of Stem Cell Sciences, Graduate School of Health Sciences, Hacettepe University, Ankara, Turkey; 2 Center for Stem Cell Research and Development, Hacettepe University, Ankara, Turkey; 3 Department of Chemistry, Faculty of Engineering, Istanbul University-Cerrahpasa, Istanbul, Turkey; 4 Department of Chemical Engineering, Faculty of Engineering, Istanbul University-Cerrahpasa, Istanbul, Turkey

**Keywords:** Breast cancer stem cells, 3D breast cancer model, Oleuropein, Metachrylated alginate (mALG), MCF-7

## Abstract

**Background:**

Cancer stem cells (CSCs) are a subpopulation of cancer cells that are believed to be responsible for tumor initiation, progression, metastasis, and resistance to conventional therapies. Oleuropein as a natural compound found in olive leaves and olive oil, has potential therapeutic effects in cancer treatment, particularly in targeting CSCs. It induces apoptosis in CSCs while sparing normal cells, inhibit proliferation, migration, and invasion, and suppress the self-renewal ability of CSCs. Additionally, oleuropein has shown synergistic effects with conventional chemotherapy drugs, enhancing their efficacy against CSCs.

**Objectives:**

This study aims to selectively target therapeutically resistant cancer stem cells (CSCs) within a heterogeneous tumor population by utilizing oleuropein (OLE) encapsulated in methacrylated alginate (OLE-mALG) within an in vivo-like microenvironment.

**Purpose:**

This study aims to target therapeutically resistant cancer stem cells (CSCs) with oleuropein (OLE) encapsulated in the methacrylated alginate (OLE-mALG) in a heterogeneous tumor population with an in vivo-like microenvironment.

**Methods:**

Co-culture of CSCs with non-tumorogenic MCF-12 A cells was performed, the 3D breast cancer model was supported with methocel/matrigel/collagen-I, and vascularization was ensured with human umbilical vein endothelial cells (HUVEC). Then, OLE-loaded methacrylated alginate microparticles (mALG) were formed by dual crosslinking in the presence of both ionic and visible light obtained with a droplet based microfluidic system. The characterization and effectiveness of the produced OLE-mALG were evaluated by the FTIR, swelling/degradation/release analysis. Before producing OLE loaded mALG microparticles, a preliminary study was carried out to determine the effective dose of OLE for cells and the duration of OLE action on MCF-7, CSCs and MCF-12 A. Subsequently, CSC viability (WST-1), apoptosis (Bcl-2, Bax, caspase-3, caspase-9), stemness (OCT3/4, NANOG, SOX2), EMT profile (E-cadherin, Vimentin, Slug) and proliferation (SURVIVIN, p21, CYCLIN D1) after OLE-mALG treatment were all evaluated in the 3D model.

**Results:**

OLE was encapsulated in mALG with an efficiency of 90.49% and released 73% within 7 h. OLE-mALG induced apoptosis through the decrease in anti-apoptotic Bcl-2 and an increase in pro-apoptotic Bax, caspase-3, and caspase-9 protein levels. While Vimentin and Slug protein levels decreased after 200 µg/mL OLE-mALG treatment to 3D breast cancer culture, E-cadherin levels increased. OLE-mALG treatment to CSC co-culture led to a decrease in proliferation by triggering p21/SURVIVIN expressions, and also resulted in an increase in stemness genes (OCT3/4/NANOG/SOX2).

**Conclusion:**

200 µg/mL OLE-loaded mALG microparticles suppressed epithelial-to-mesenchymal transition by suppressing Vimentin and Slug protein levels, and increased E-cadherin levels in the 3D breast cancer model we created with CSCs, MCF-12 A and HUVECs. This complex system may allow the use of personalized cells for rapid drug screening in preclinical studies compared to animal experiments. OLE-mALG showed apoptotic and metastasis suppressive properties in cancer cells and it was concluded that it can be used in combination with or alternatively with chemotherapeutic agents to target breast cancer stem cells.

**Graphical abstract:**

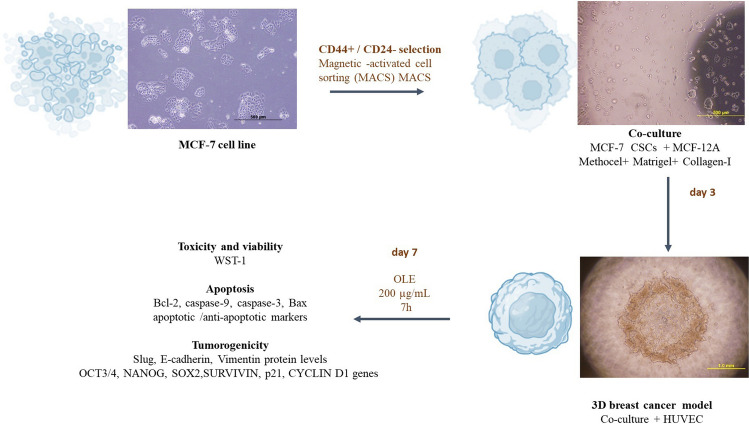

**Supplementary Information:**

The online version contains supplementary material available at 10.1007/s40199-024-00512-3.

## Introduction

Breast cancer stem cells (CSCs), representing only a small portion (0.1-1%) of the heterogeneous tumor population, exert significant influence on tumorigenesis, metastasis, drug resistance, and tumor recurrence. Their resemblance to normal stem cells underscores their significance in cancer progression and therapeutic resistance as they have self-renewal ability and maintain a non-proliferative state [1]. Therefore, the currently used anti-cancer drugs/metabolites are generally insufficient in targeting cancer stem cells (CSCs) and in preventing relapse/distant metastasis [1]. Therefore, the currently used anti-cancer drugs/metabolites are generally insufficient in targeting cancer stem cells (CSCs) and in preventing relapse/distant metastasis. Developing a patient-specific therapy offers a powerful response in anti-cancer treatments. In recent years, there has been increasing interest in studying the effectiveness of various combinations of anti-cancer agents using patient-derived tumor cells within a three-dimensional (3D) environment that replicates the conditions of the tumor [2]. Djomehri et al. created a breast cancer organoid model with non-tumorogenic MCF-10 A epithelilal cells and MDA-MB-231 cell line. They reported that the expression of mammary gland progenitor markers and development of lumen/secondary acini structures were observed. This 3D structure was able to mimic tissue-like structure in-vivo using ultra-low attachment plates and matrigel [3]. In another study, addition of methylcellulose (methocel) and matrigel to the culture medium has shown to support the uniformly shaped spheroid formation of breast cancer cells [4].

Multiple studies have elucidated that the anti-angiogenic secoiridoid oleuropein, derived from olive oil and olive leaves, exerts a dose-dependent and irreversible inhibition on the proliferation, invasion, and replication of cancer cells, in contrast to its impact on normal cells [5]. OLE or hydroxytyrosol (HT) leads to blocking various molecular pathways involved in human tumorigenesis and eliminating the anti-apoptotic mechanism in CSCs and cancer cells. Han et al. showed that HT and OLE, decreased cell viability and proliferation of MCF-7 by arresting cells in G1/S phase and, triggered cell apoptosis [6]. In another study, OLE treatment resulted in a significant decrease in cell viability of MCF-7 and MDA-MB-231 cells by triggering mitochondrial pathway-mediated apoptosis, leading to S-phase arrest in the cell cycle. Moreover, apoptotic effect of OLE was detected selectively in cancer cells with no effect on non-tumorigenic MCF-10 A epithelial cells [7]. . Erdogan et al. reported that apigenin flavonoid in olive oil inhibited cell survival dose-dependently by upregulation of p21 and p27 in prostate CSCs and decreased the migration of CD44 + CSCs [8]. Asgharzade et al. showed that OLE induced apoptosis in MCF-7 and MDA-MB-231 cells by causing oncomiR and anti-apoptotic gene downregulation, upregulation of tumor suppressor miRNA and pro-apoptotic genes [9]. Tezcan et al. found that the combination of olive leaf extract and bevacizumab (avastin) resulted in a significant reduction in tumor size, vascularization, and migration of glioblastoma cells [10]. Although there are numerous studies on the effects of OLE in various tumor types and CSCs, this mechanism has not been fully explored and detailed with regards to breast CSCs.

Due to the hydrophilic character of anti-proliferative and antioxidant OLE, researchers have attempted to produce more stable hydrophobic derivatives and/or develop suitable drug delivery systems. In a study, researchers demonstrated that OLE inside lipid carriers and its semisynthetic acetyl-derivatives reduced LPS-induced inflammatory response in murine peritoneal macrophages without OLE degradation [11]. OLE encapsulated in nanostructured lipid carriers has been shown to protect and increase the antioxidant effect of OLE in A549 and CuFi-1 lung epithelial cells [12]. In recent years, microfluidics has been a powerful tool for the manipulation and encapsulation of cells and drugs in microgels. Creating biopolymer-based hydrogel scaffolds that can mimic the ECM with their biochemical and physical properties is promising in tissue engineering applications. Therefore, alginate has also been a suitable candidate for encapsulation of cells and drugs in alginate hydrogels due to its mild gelling conditions and biocompatible properties. It is also non-toxic, biodegradable, hydrophilic, non-allergenic and inherently devoid of immunogenicity with a wide range of applications. In addition, to improve various properties of alginates, unsaturated groups are added to the main chain and crosslinking is then carried out under light through these groups. In this regard, studies on the production of microparticles of different biomaterials with fluid systems have become widespread. Sheikhi et al. produced microfluidic hydrogels from microbeads using a naturally derived based protein [13]. In this study, 20% w/v 50–100 kDa methacrylated gelatin (GelMA) was used and 140 μm microbeads were obtained using a flow-oriented system in HFE 7500 continuous phase medium. An et al. developed a microfluidic-based approach for continuous encapsulation of mesenchymal stem cells (MSC) in single-cell alginate microgels to be used in bone tissue engineering applications [14]. This enabled the scalable production of cell-loaded microgels while maintaining the viability and functionality of the encapsulated cells. In addition, osteogenesis and hydrogel matrix mineralization of MSCs encapsulated in alginate microgels were significantly accelerated. In another study, non-spherical, cytocompatible, degradable, monodisperse alginate microgels were formed for chondrocyte encapsulation by the microfluidic technique using oxidized methacrylate alginate [15]. Microgels prepared in the microfluidic system using LED UV light for crosslinking were synthesized in situ as crosslinked.

## Methods

### Cell culture

The MCF-7 cell line (ATCC) was cultured in RPMI 1640 medium (Gibco) containing FBS, L-glutamine, and penicillin/streptomycin, and maintained in an incubator set at 37 °C with 5% CO_2_. Non-tumorigenic epithelial cell line MCF-12 A (ATCC) was cultured in DMEM/F12 medium (Gibco) supplemented with Nu serum (Corning), ITS Premix (Corning), penicillin/streptomycin (Sigma) and L-glutamine (Sigma) and incubated at 37 °C with 5% CO_2_. Human umbilical vein endothelial cells (HUVEC, Lonza) were cultured in EGM-2 containing FBS, penicillin/streptomycin and L-glutamine at 5% CO2 and 37^o^C culture conditions. The cell culture medium was changed every two days for all cultures.

### Isolation and characterization of cancer stem cells from MCF-7 cell line

The isolation of CSCs from confluent MCF-7 cells was performed using the Magnetic-Activated Cell Sorting (MACS) method with anti-CD24 and anti-CD44 (Miltenyi) magnetic beads, following the manufacturer’s instructions. After centrifugation at 300xg for 10 min, CSCs were resuspended in FACS buffer. They were then incubated for half an hour with anti-CD24-PE and anti-CD44 FITC antibodies. Subsequently, the characterization of CD44^+^/CD24^−/low^ CSCs was performed using the BD AccuriTM C6 Flow cytometer (BD Bioscience).

### Co-culture and generation of 3D breast cancer model

A medium was prepared to support mammosphere formation properties of CSCs, containing EGF (Sigma), bFGF (Sigma), B-27 (Sigma), L-glutamine (Sigma), and penicillin/streptomycin in RPMI 1640 medium (Gibco). When isolated CSCs were co-cultured with MCF-12 A cells, the culture media and cells prepared for both cell types were mixed in a 1:1 ratio. Methylcellulose (Methocel® MC, Fluka), Matrigel (Sigma), and Collagen-I (Advanced Biomatrix) were added to the created suspension to support the formation of a 3D structure, and the cell culture was maintained for three days. To promote vascularization within the organoid-like structure, HUVECs (Lonza) were introduced into the co-culture, extending the cell culture period by an additional three days. Medium changes were performed by removing half of the old medium and adding an equal amount of fresh medium to sustain the culture. The cell population of the 3D culture was assessed by flow cytometry. Cells were harvested using 3 mg/mL collagenase-I (Sigma) and trypsin, resuspended in FACS buffer solution, and then labeled with anti-human anti-CD44-FITC and anti-CD-24-PE antibodies for half an hour. CD44 + and CD24- cells were identified using the BD AccuriTM C6 Flow Cytometer (BD Bioscience).

### Evaluation of the effect of OLE on cells within the 3D breast cancer model

Western Blot was performed to assess the effect of OLE on tumorigenic MCF-7 cells, CSCs and on non-tumorigenic MCF-12 A cells. After cells were harvested and tripsinized, total protein extraction was performed by RIPA lysis buffer (Serva) with protease inhibitor cocktail (Roche). Concentration of the extracted total protein was determined by BCA Assay Kit (Thermofisher). 30 µg of total protein per sample was loaded into the wells of the 10% separating gel. After the run, Trans-Blot Turbo (Bio-Rad) was used for membrane transfer (Advansta). Following washing and blocking with 5% non-fat milk powder (TBS-T), Caspase-3 (Abcam), Caspase-9 (Biorbyt), Bax (Biolegend), Bcl-2 (Biolegend), GAPDH (Biolegend), HRP-anti rabbit, and HRP-anti-mouse (Biolegend) antibodies were used. Each antibody binding was performed by the following steps: blocking, binding of the primary antibody, washing with TBS-T, binding of the secondary antibody, washing, and image acquisition using ECL (Advansta). Protein bands were visualized by ProteinSimple.

### Synthesis of methacrylated alginate (mALG)

Briefly, 2.5% w/v alginate (low viscosity alginate, Sigma) was weighed and prepared. Then, it was dissolved with distilled water (100 mL) to form a homogeneous solution. The same volume of methacrylic anhydride (MA, purity ≥ 94%, Sigma) was slowly added into the completely dissolved alginate. The reaction vessel was then covered with foil. The reaction pH control was done with 5 N NaOH, and it was adjusted to 7. The reaction continued in this way for three days. pH was frequently checked throughout the reaction process. Then, mALG was precipitated with ethanol (500 mL) and taken as a solid. Then, precipitated mALG was resolved in DI water. Solution was taken in dialysis membrane. It was dialyzed by using DI water for 7 days. After the dialysis process, the solution was taken into a beaker, frozen and freeze-dried using TeknoSEM brand lyophilizer device for two days.

### Characterization of mALG

The unmodified and modified alginate were characterized by using Fourier Transform Infrared (FTIR) and Proton Nuclear Magnetic Resonance Spectroscopy (^1^H NMR) analyzes. A Jasco FT/IR-6700 spectroscopy was used for FTIR and Varian UNITY INOVA instrument was used ^1^H NMR for characterization studies.

### Preparation of OLE loaded mALG microparticles

Microparticles production was carried out with the microfluidic device. The microfluidic device design was prepared using PDMS material as a droplet-based flow-oriented system. It was prepared from PDMS material, attached to the microscope glass. In the designed system, uniform sized microparticles were produced with the oil-water emulsion method. OLE-loaded mALG microbeads were obtained by using the following procedure. Briefly, 0.2% (w/v) of OLE was weighed and transferred into a glass tube. Then, 1 mL of photoinitiator solution in DPBS (TEA, 1.875% (w/v), VC 1.25% (w/v) and Eosin Y disodium salt (0.5mM)) was added. It was left for 1 h under constant stirring at 250 rpm at 50^o^C. Afterwards, 10% by weight of mALG was added and allowed to stir for an additional 1 h under same conditions. To prepare the OLE loaded mALG beads, the OLE loaded mALG solution was used as a dispersed phase, while 0.5% w/w Span80 in Mineral oil was used as a continuous phase to prepare a water-in-oil emulsion. Both phases (continuous phase and dispersed phase) were injected separately into the inlet microchannels of the one step emulsification microfluidic device using syringe pumps (Harvard Apparatus PHD 2000, Holliston, MA). The syringes were connected to the luer-stub inlets using polyethylene PE-5 tubing with an outer diameter of 1.32 mm and an inner diameter of 0.86 mm. The flow rate of the continuous phase was kept constant at 100 µL/min, while the flow rate of the dispersed phase was adjusted to obtain droplets of ~ 186 μm. Continuity in droplet formation was observed when the dispersed phase was 3.33 µL/min and the continuous phase was 1.66 µL/min. Then, microparticles were produced and collected in an eppendorf tube. Then, they were crosslinked ionically by using 1 M CaCl2 and were separated from the oil. Finally, the separated microparticles were cured by using visible light, a small LED light source (VALO Light Curing Device, Ultradent, USA) for 240s to make microparticles chemically crosslinked.

### Characterization of OLE loaded mALG

The structural analysis of mALG and OLE-loaded mALG microparticles were carried out by using Fourier Transform Infrared (FTIR) analysis. FTIR spectra were recorded on Jasco FT/IR-4600 using ATR adapter in the wavelength range of 400–4000 cm^−1^. The dimensions and surface pore morphology of mALG and OLE-mALG microparticles were investigated by scanning electron microscopy (SEM) using ESEM-FEG (JEOL JSM 5600.). ImageJ software was used to evaluate the sizes of microparticles.

### Drug release studies

Samples were prepared as microparticles, and total drug content was calculated as a function of sample weight and designed weight ratio. Following this, drug release behavior was tested by immersion in 20 mL of phosphate buffered saline (PBS) solution at 37 °C. At the time points, 3 mL of PBS was removed from the solution and the system was refilled with 3 mL of fresh PBS. The absorbance of UV light of the received PBS solution was measured at 250 nm which is a characteristic absorbance value for OLE with a UV-vis spectrophotometer (VALO Light Curing Device, Ultradent, USA). The concentration was then interpreted via an absorbance-concentration calibration curve ranging from 5 to 100 ppm. The calibration curve was calculated as y = 0.0055x + 0.006 and R2 = 0.9996. Here, Y represents the absorbance value of the solution at 250 nm while X represents the OLE concentration (ppm). With the help of the obtained calibration curve, cumulative drug release curves were drawn over time. Experiments were carried out in triplicate.

The OLE loading of the microparticles was estimated according to the following method [16]. The particles were immersed into 20 mL of PBS and broke were broken the particles completely and incubated over 48 h. The absorbance of the supernatant solution was measured by using a UV-Vis spectrophotometer (the specific absorbance value of OLE). The OLE loading was determined by using the standard curve of OLE release and was calculated by the following equation (Eq. 1):

1 $$Drug \ Loading \ Efficiency \left(\%\right)=\frac{Amount \ of \ maximum \ OLE \ release }{Initial \ amount \ of \ OLE \ containing \ beads} \times100$$

Equation 1. Calculation of OLE loading efficiency.

### Physical properties of mALG and OLE loaded mALG

To determine the swelling behavior of OLE loaded mALG microparticles, the prepared hydrogel was pipetted into silicone molds and was exposed to visible light for cross-linking. It was then frozen and lyophilized. The samples prepared for measurement were weighed dry and incubated in PBS solution at 37^o^C. It was then weighed at certain time intervals (1 h, 3 h, 5 h, 7 h and 24 h). Samples were prepared as 3 pieces, *n* = 3 and mALG-based samples without OLE were accepted as controls. The swelling ratio of the microparticles was calculated using the equation below (Eq. 2). Here, W0 represents the initial weight and Wt indicates the wet weight of the samples.

2 $$Swelling \ Ratio \left({\%}\right)=\frac{Wt-W0}{W0} \times100$$

Equation 2. Calculation of the swelling ratio of the microparticles.

The degradation behavior of the mALG and OLE loaded mALG microparticles were evaluated by immersing them in completely dry and pre-weighed cylindrical samples in PBS solution containing 10 µg/mL of collagenase at 37^o^C in 24 h. After this process, the samples were removed from incubation and lyophilized. Weight reduction of samples was determined by calculating the dry mass of the sample before and after incubation. All specimens were measured as triplicate.

### Effect of OLE-mALG on cell viability

Water Soluble Tetrazolium-1 (WST-1) assay (Roche) was conducted to determine the cytotoxic effect of OLE and to assess cell viability in the created 3D breast cancer model cells. For viability assessment, 10 µL of WST-1 was added to cells cultured in a 96-well plate with 100 µL of medium, in both groups treated and untreated (control) with OLE. After a 2-hour incubation period, the absorbance value (OD) for each sample was measured with a microplate reader at a wavelength of 450 nm.

### Effect of OLE-mALG on tumorigenicity and apoptosis

RT-qPCR and Western Blot experiments were carried out to evaluate the anti-tumorigenic and apoptotic properties of CSCs following OLE-mALG treatment.“Initially, the organoid-like structure was dissociated into a single-cell suspension using collagenase (Sigma) and trypsin (Sigma). The cells were then harvested, and total protein extraction for Western Blot Analysis was conducted using RIPA lysis buffer (Serva) and a protease inhibitor cocktail (Roche). The following steps were performed as described in the previous section. The protein levels were assessed in both mALG-OLE treated and untreated (control) cell groups using anti-human anti-Slug (ST Johns Lab.), anti-Vimentin (Biolegend), anti E-cadherin (Biolegend), anti-GAPDH (Biolegend), and HRP-anti-mouse antibodies. Gene expression analysis (NANOG, OCT3/4, SOX2, SURVIVIN, CYCLIN D1, P21, GAPDH, Table 1) was performed by RT-qPCR (LightCycler® 480 II, Roche, Germany). Total cellular RNA was isolated using the “HibriGen RNA Isolation Kit” (Turkey). The isolated RNA concentration was measured at 260 nm using the NanoDrop ND-1000 spectrophotometer (Thermo Fisher Scientific Inc., USA) and the A260/A280 ratio was used to measure the RNA quality. Complementary deoxyribonucleic acid (cDNA) synthesis was performed from RNA samples using the High-Capacity cDNA Reverse Transcription Kit ProtoScript® II First Strand cDNA Synthesis Kit (NEB). Quantitative PCR condition: 10 min denaturation step at 95°C; A total of 45 cycles were set as a PCR step at 95°C for 10 seconds, at 60°C for 30 seconds, at 72°C for 1 second, and at 40°C for 30 seconds as a cooling step. Three biological replicates were run for each different experimental condition and three technical replicates were performed for each sample. Crossing point (Cp) or threshold cycle (Ct) value was calculated for target and reference genes using LightCycler® 480 II software.

**Table 1:** Primer sequences for detection of mRNA levels

GAPDH	F 5’-GTTGGAGGGAAGGTGAAGTTC-3’ R 5’-TGTGTCTATCTACTGTGTCCCA-3’
SURVIVIN	F 5’-CCAGTGTTTCTTCTGCTTCAAG-3’ R 5’-CAAACTGCTTCTTGACAGAAAGG-3’
CYCLIN D1	F 5’-GTGTCCACTTCAAATGTGTGC-3’ R 5’-AGCGGTCCAGGTAGTTCA-3’
OCT3/4	F 5’-GTTGGAGGGAAGGTGAAGTTC-3’ R 5’-TGTGTCTATCTACTGTGTCCA-3’
NANOG	F 5’- GCTGGTTGCCTCATGTTATTATGC-3’ R 5’- CCATGGAGGAAGGAAGAGGAGAGA-3’
SOX2	F 5’-GTACAACTCCATGACCAGCTC-3’ R 5’-AGCGGTCCAGGTAGTTCA-3’
P21	F 5’-CTGCCCAAGCTCTACCTTC-3’ R 5’-GTCCACATGGTCTTCCTCTG-3’

### Statistical analysis

SPSS Inc. was used for both parametric and non-parametric statistical analyses. t-tests and ANOVA analyses were conducted to compare different experimental groups. Data with a p value less than 0.05 were considered statistically significant. Unless otherwise stated, each sample was run three times.

## Results

### Generation and characterization of 3D breast cancer organoid-like structure

**Fig. 1:**
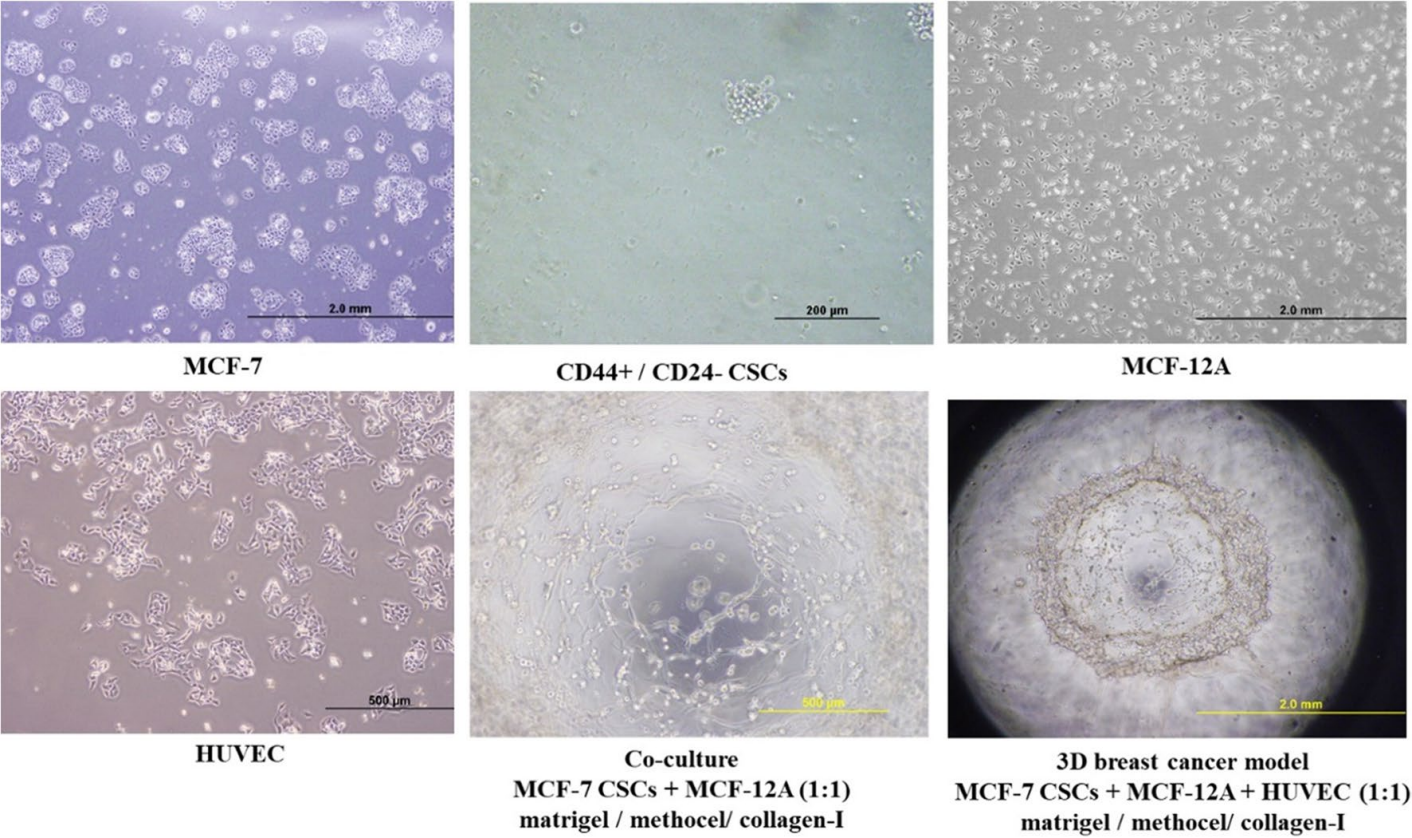
Representative images of cells in culture and the 3D breast cancer model. Scale bar: 200 μm, 500 μm, 2.0 mm

The cells to be cultured in the 3D culture model were initially morphologically characterized using an Olympus CKX41 light microscope. A varying range of CD44+/CD24-/low CSCs was obtained from 8 × 10^5^ to 10^6^ out of 50 × 10^6^ MCF-7 cells through MACS (Magnetic-Activated Cell Sorting) on the 7th day of culture. Characterization of these cells was performed using flow cytometry with 100% CD44 + and 1% CD24 + phenotype (Fig. Suppl. 1). MCF7 cells exhibited adherent characteristics, while CD44+/CD24-/low CSCs are observed in a suspended state and formed colonies (Fig. 1). CSCs and MCF12A cells (at a 1:1 ratio) were cultured by mixing with Matrigel, Methocel, and Collagen-I components (day 0). Subsequently, vascularization of the 3D structure was achieved by co-culturing with HUVECs (Human Umbilical Vein Endothelial Cells) on the 3rd day (Fig. 1). The diameter of the colonies formed with CSCs was determined to be 114.3 ± 25.7. The diameter of the 3D model created with CSC, MCF-12 A and HUVEC, was determined to be 2.0 ± 0.4 mm. The presence of two distinct populations with CD44+/CD24- characteristics within the created 3D structure was determined using flow cytometry (Fig. Suppl. 1).

To understand the effect of OLE on MCF-7, CSCs and MCF-12 A, we selected different concentrations (0, 200 µg/mL) and time intervals (0, 4 and 7 h) of OLE based on the literature (Fig. Suppl. 2). According to the preliminary studies, after 200 µg/mL OLE application, apoptotic caspase-3, caspase-9 and Bax protein levels gradually increased with application time both for MCF-7 and CSCs (Fig. Suppl. 2a and 2b). This data suggests that a 200 µg/mL OLE application for 7 h could be more effective in eliminating the MCF-7 cell line and the CSCs. In contrast, Western Blot experiments conducted following OLE application to MCF-12 A non-tumorigenic epithelial cells did not reveal an increase in the expression of apoptotic markers, in line with the literature (Fig. Suppl. 2c) [17].

### Characterization of mALG and OLE-loaded mALG

**Fig. 2:**
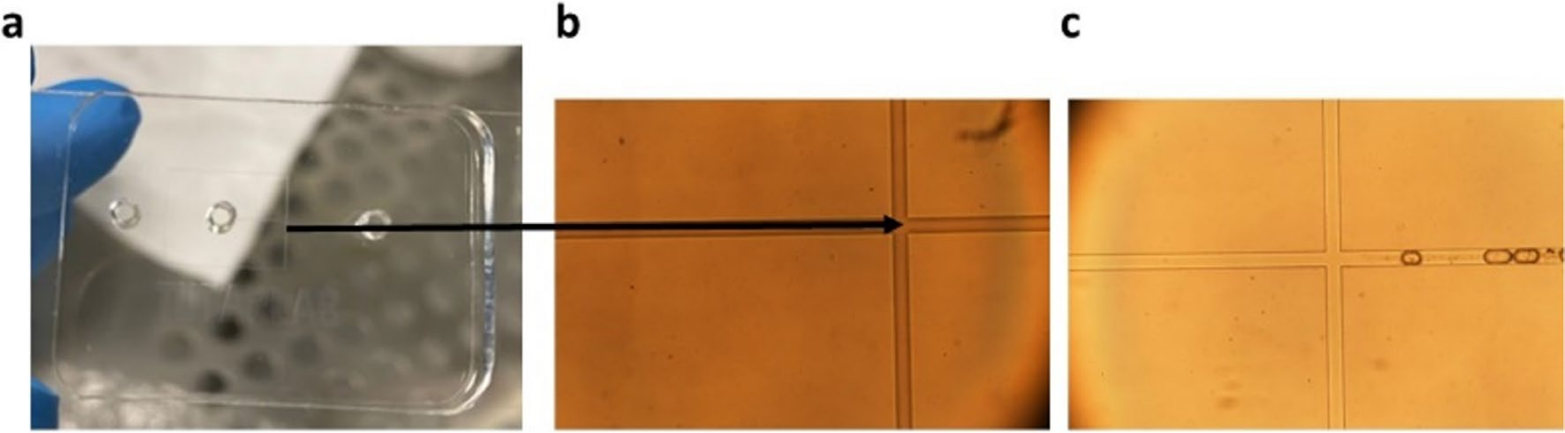
**a**-**b** Droplet based microfluidic device. **c **Production of OLE-mALG microparticles

Droplet based microfluidic device was designed and fabricated to produce OLE encapsulated mALG microparticles (Fig. 2). The droplet-based microchip, where the OLE-mALG were synthesized in a droplet was generated by using PDMS mold. OLE encapsulated mALG microparticles were produced by employing the water-in-oil emulsion-based method. Here, the OLE loaded mALG solution was used as a dispersed phase, while 0.5% w/w Span80 in mineral oil was used as a continuous phase to prepare a water-in-oil emulsion.

The microparticles were produced and collected in eppendorf tube, which they were then crosslinked ionically by using 1 M CaCl_2_ and were separated from the oil. Finally, the separated microparticles were cured by using visible light, a small LED light source (VALO Light Curing Device, Ultradent, USA) for 240 s to chemically crosslink the microparticles.

**Fig. 3:**
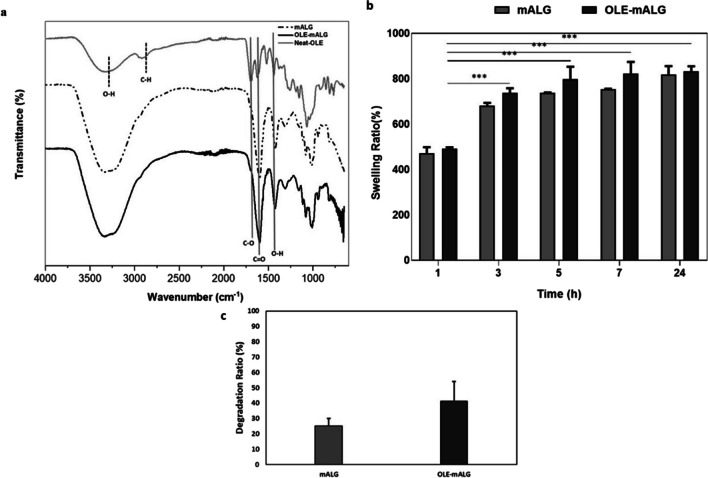
**a **FTIR spectra of mALG, OLE-mALG and neat OLE. **b **Swelling ratio of mALG and OLE-mALG beads. **c **Degradation ratio of mALG and OLE-mALG beads. Data are shown as mean ± SD, *n* = 3. The results were statistically analyzed using one-way ANOVA. *p < 0.05, **p < 0.01; ***p < 0.001

FTIR analysis was performed to prove that we encapsulated the OLE into the mALG-based hydrogel system (Fig. 3a). In the FTIR spectrum of pure OLE, the broadband at 3305 cm^−1^ may denote the O-H stretch, while 2900 cm^−1^ can represent C-H stretch vibration signals. Moreover, ~ 1700 cm^−1^ and ~ 1615 cm^−1^ can be attributed to the characteristic vibration of the two carbonyl groups in the OLE [18]. The signals of functional groups C = C and O-H of the OLE appeared as a stretch at 1517 cm^−1^ and a bending vibration at 1440 cm^−1^, respectively [18]. According to the literature, 1440, 1575 and 1625 cm^−1^ are the characteristic peak values of OLE [19]. Consequently, when the FTIR peaks of pure mALG-beads and OLE loaded mALG beads were examined, the sharpening of the peaks at 1700, 1610 and 1425 cm^−1^ in OLE loaded mALG was explained by carbonyl groups and O-H group of OLE, respectively. The reason for this low sharpening in the obtained peaks can be explained by the low absorbance values of the prepared microparticles caused by the small amount of OLE determined in the formulation [20].

Appropriate degradation behavior of biomaterials is a key factor for their applications in tissue engineering and drug delivery systems [21]. In this context, the beads were incubated in PBS medium at 37^o^C in the presence of collagenase enzyme and the degradation rate in 24 h was measured (Fig. 3b). When the obtained results were examined, it was seen that the degradation rates of mALG and OLE loaded mALG beads were 21% and 45%, respectively. To examine the swelling behavior of the hydrogel-based beads, we incubated the lyophilized samples in PBS medium at 37^o^C and measured the swelling values at certain time intervals (1 h, 3 h, 5 h, 7 h, 24 h). Figure 3c shows that both hydrogel-based beads exhibited high swelling behavior. No significant difference was observed in swelling rates. This improved swelling rate may be related to the hydrophilic nature of the sodium alginate biopolymer.

**Fig. 4:**
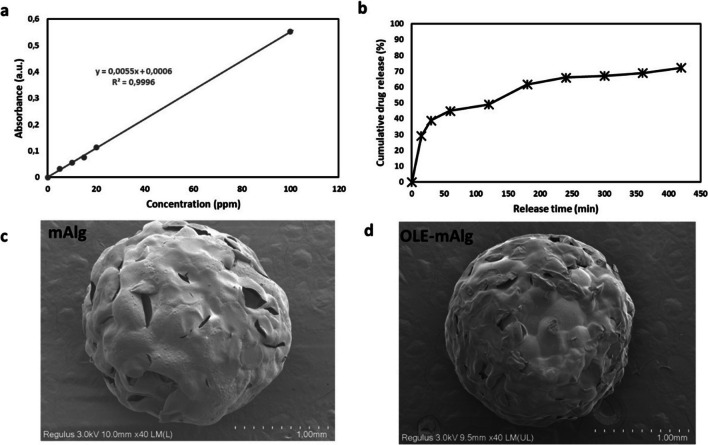
**a **The calibration curve of different concentrations of OLE. **b **OLE release profiles of OLE-mALG beads. **c** and **d **SEM images of mALG and OLE-mALG beads, respectively

An in vitro release study was performed to investigate the drug release behavior of mALG beads. OLE release profiles from mALG beads were evaluated in PBS at 37 °C (pH = 7.4). Plots for cumulative OLE release profiles as a function of time are shown in Fig. 4a. Accordingly, it was determined that OLE was loaded into the mALG matrix with 90.49% efficiency. Drug release data for mALG beads showed that 45% of OLE was released within the first hour and approximately 73% of OLE was released within 7 h (Fig. 4b). Thus, it was noted that a controlled release took place. The prepared mALG and OLE-mALG particles were characterized morphologically using the SEM images provided in Fig. 4c-d to determine their average particle sizes and surface features [22]. It is evident from these images that an explicit spherical shape is achieved for each micro particle. The average sizes of microspheres were calculated as 186 ± 27,30 μm and 184 ± 21,90 μm for mALG and OLE-mALG, respectively. In this case, it was determined that the presence of OLE did not affect the size of the beads. Additionally, while hierarchical closed porosity is observed on the surface of mALG particles, the presence of OLE results in a larger closed pore morphology on the particle surface.

### Effect of m-ALG particles on cell viability, apoptosis and tumorigenicity

**Fig. 5:**
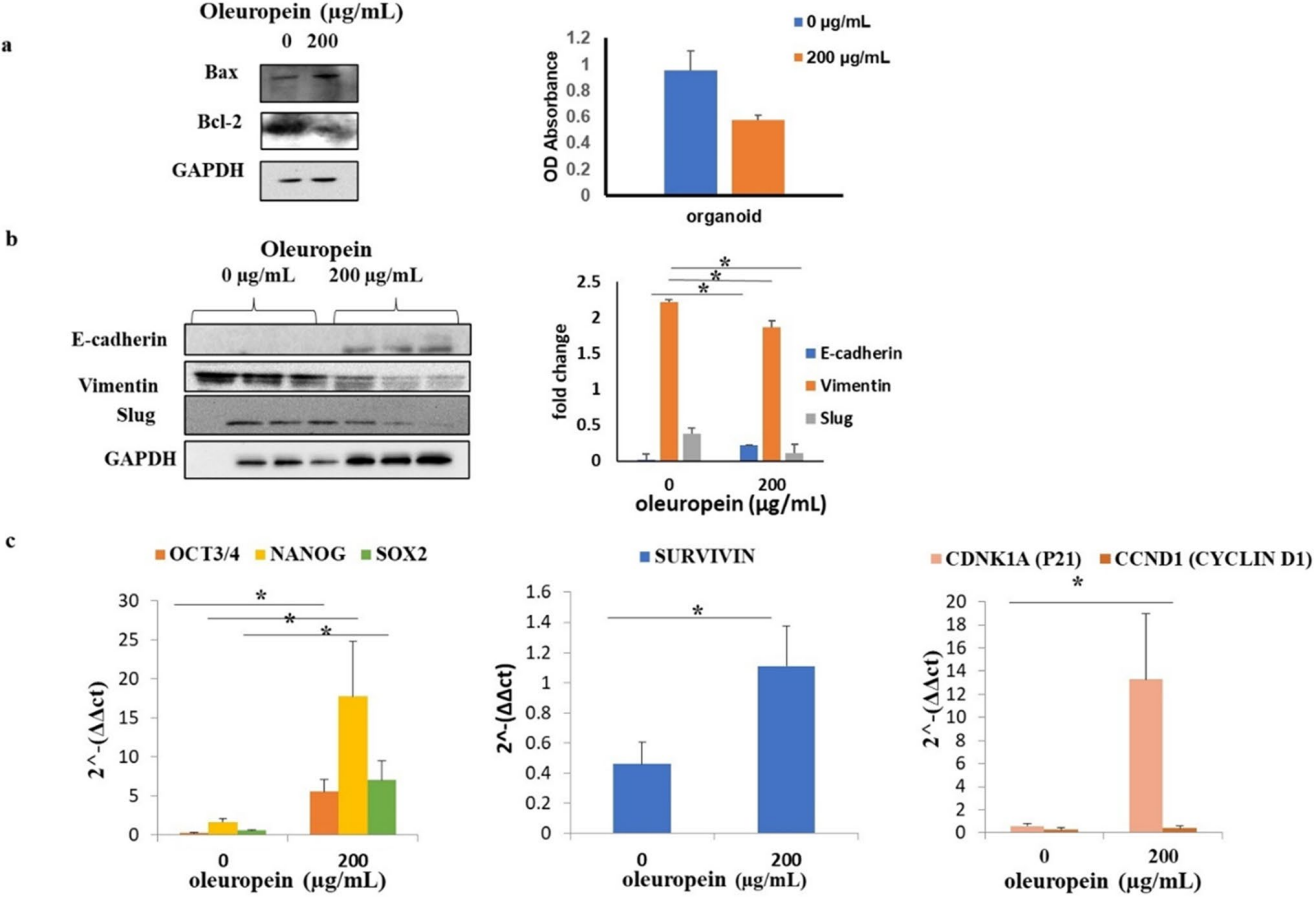
Determination of cell viability and tumorigenic potential of organoid cells after OLE-mALG treatment (200 µg/mL, 7 h). **a** pro-apoptotic Bax and anti-apoptotic Bcl-2 protein levels obtained by Western Blot (**b**) cell viability assessment by WST-1 determination in organoid model with and without OLE-mALG. Before administration: control group, after administration: targeted organoid model. **c** Western blot analysis and protein levels before/after OLE-mALG treatment. **d** Gene expressions before / after OLE-mALG treatment Data are shown as mean ± SD, *n* = 3. The results were statistically analyzed using one-way ANOVA. *p < 0.05

Although OLE-mALG treatment triggered an increase in the pro-apoptotic Bax protein level, anti-apoptotic Bcl-2 protein level was decreased in 3D breast cancer model (Fig. 5a). In parallel to this observation, WST-1 analysis demonstrated a decrease in cellular viability compared to the control group after OLE-mALG application. Following the application of 200 µg/ml OLE, cell viability was determined to be 67% compared to the untreated group, as indicated by WST-1 analysis. Cells decreased their Vimentin (1.3- fold) and Slug (2-fold) protein levels after OLE-mALG (Fig. 5b, *p* < 0.05, *n* = 3). In contrast, E-cadherin protein level was approximately 15-fold higher after OLE-mALG treatment compared to control (Fig. 5b, *p* < 0.05, *n* = 3). For the determination of the tumorigenic potential of cells after treatment with OLE-mALG, we performed RT-qPCR analysis (Fig. 5c). After OLE-mALG treatment, expression of pluripotency genes (OCT3/4, NANOG, SOX2) were increased (27-fold, 10-fold, 4-fold, respectively, *n* = 3, *p* < 0.05). Similarly, SURVIVIN (2-fold) and p21 (22-fold) mRNA levels were also increased with OLE-mALG treatment in cells (*p* < 0.05, *n* = 3). However, CYCLIN D1 mRNA levels remained unchanged (*p* > 0.05, *n* = 3).

## Discussion

Epidemiological studies reveal that the dietary intake of phenolic compound oleuropein (OLE) which is found in olives and olive leaves reduces the risk of many diseases [23]. It has been supported by various studies that OLE acts selectively in targeting tumor cells [24]. Although it has been thought that OLE exhibits this anti-tumorigenic effect through various signaling pathways related to cell proliferation, survival, invasion and metastasis, its mechanism of action has not been detailed. The effect of OLE on proliferation, tumor development and metastasis of breast cancer cells was investigated in in vitro experiments and animal models. In this study, we investigated whether it could target cancer stem cells in tissue-like 3D structure by loading the antioxidant OLE into the microparticle via a microfluidic system.

The development of personalized treatment approaches and 3D cancer organoid models holds immense promise for advancing cancer research and therapy. Personalized treatment strategies, tailored to individual patient profiles, have the potential to improve treatment efficacy and minimize adverse effects. Concurrently, the establishment of 3D cancer organoid models allows for more accurate representation of *in vivo t*umor behavior, fostering a deeper understanding of cancer progression and drug responses. The integration of personalized treatment and 3D cancer organoid models provides a valuable platform for preclinical testing, drug screening, and therapeutic optimization, ultimately bringing us closer to more effective, patient-specific cancer treatments. Culture systems incorporating 3D mammary epithelial cells (MCF-10 A and MCF-12 A) are becoming common for the study of mammary gland biology. The interaction of normal and cancerous breast epithelial cells and endothelial cells is also used in studies of tissue development, angiogenesis, and metastasis. Studies on the incorporation and co-culture of endothelial cells into 3D breast spheroids that will form vascular structures have shown that the formed 3D structure contains endothelial tube-like vascular networks [25]. Unlike MCF-7 cells, MCF-12 A cells are non-tumorigenic and ER-negative, and it has been demonstrated in the literature that they mimic the mammary gland-like structure by forming lumen and acini in MCF7 cells in matrigel-based 3D culture systems [26]. By developing this structure, we aimed to demonstrate the effect of OLE and OLE-mALG in the in the breast cancer model. It has been shown that CSCs maintain their cancer-initiating potential and self-renewal capacity and lead to tumorigenesis and drug resistance in malignant tumors *in vivo.* Moreover, OLE suppresses metastasis in triple negative breast cancer cells, triggers cell cycle arrest and apoptosis with an increase in ROS production and eliminates CSCs. In our study, we aimed to determine whether OLE would directly target resistant cancer stem cells rather than healthy surrounding cells in the 3D breast model we have created of ER + MCF-7 CD44+/CD24- (CSC) cells and how OLE would affect their stemness, proliferative and metastatic properties of this population [27].

In this study, we created a breast CSC organoid-like model with non-tumorogenic breast epithelial cells, collagen, matrigel and methocel, for the first time [3]. In this study, we aimed to evaluate tumorigenicity through targeting the 3D structure with OLE (Fig. 1, Fig. Suppl. 1). MCF-7 derived CSCs were characterized by their spheroid formation potential and their CD44+/CD24- cell surface marker profile similar to the literature (Fig. 1, Fig. Suppl. 1a). In addition, the distribution of CD44+/CD24- population within the 3D structure was determined (Fig. Suppl. 1b).

We determined the effective OLE concentration and time after the western blot analysis (Fig. Suppl. 2) In our preliminary study, consistent with the literature, OLE exhibited anti-tumor properties in MCF7 and CSC cells (Fig. Suppl. 2a and 2b)., while the expression of apoptotic markers was not detected in non-tumorigenic MCF12A cells (Fig. Suppl. 2c). In the case of MCF7 and CSC cells, it was found that a 7-hour treatment with 200 µg/mL OLE was the most effective condition leading to an increase in apoptotic markers (Caspase-3, Caspase-9 and Bax) [17]. Therefore, for the ongoing studies, 200 µg/mL OLE was applied to the 3D model by loading it into methacrylated alginate microparticles over a 7 h period.

Droplet-based microfluidic devices are one of the most widely used methods to produce both droplets and micro/nanoparticles. It is possible to prevent cross-contamination, reduce sample loss and shorten diffusion times with droplet-based microfluidic systems. As such, droplet-based microfluidic was preferred in our study and microparticles were obtained from drug-loaded alginate-based biomaterials. OLE was loaded into alginate before determining the effects of OLE on tumorigenic properties in an organoid-like model. Briefly, OLE-mALG microparticles with good monodispersity and shape consistency were produced for the first time by photocrosslinking method in the presence of triple photoinitiators (VC/TEA + Eosin Y). Afterwards, we performed the structural characterization of obtaining OLE-loaded mALG beads by FTIR analysis (Fig. 3a). The results obtained with the characterization studies gave detailed results about the properties of the prepared microparticles and OLE-loaded microparticles. As known from the literature, surface porosity is a critical feature of drug encapsulation [28]. Accordingly, drugs can fill the cavities on surfaces with either open or closed pore morphology. In this study, it is evident that smaller-sized pores observed on the surface of mALG particles are not present on the surface of OLE-mALG samples. The reason for this, as clearly discernible from SEM images, is the filling of these pores with OLE. Hydrogels are widely used in drug delivery systems due to their high water content that allows enhanced drug permeability. Furthermore, hydrogels can control drug release due to their response to internal and external stimuli such as swelling or degradation. It has been proven in the literature that methacrylate alginate slows down the rate of degradation [29]. It is also well established that the degree of crosslinking slows down the degradation [21]. Considering all this, Fig. 3c shows that the beads had a controllable degradation behavior, and the OLE molecule had an accelerating effect on the degradation. Thus, the beads obtained will not degrade before the drug release is completed, nor will they suddenly release all the drug. Moreover, it will degrade after the release of the drug in the specified time and will not leave a toxic effect on the body. Controlled drug release is a very important parameter for an effective cancer treatment. In burst releases, the drug may have a toxic effect, and thus undesirable side effects may occur. We evaluated the release behavior of OLE-mALG beads in 37^o^C PBS medium before performing the release in the breast cancer model. The results clearly demonstrated the remarkable role of the double crosslinked structure and the alginate formulation in preventing burst release. Indeed, the shape consistency of the prepared microparticles was found to be much better than the production technique using the traditional ionic crosslinking method, where the crosslinker liquid flow can cut and deform the alginate droplets and the deformation is preserved, resulting in low shape consistency. An advanced ionic crosslinking method by inducing divalent cations in alginate microdroplets was also used in our study to improve shape consistency and material homogeneity. In this sense, an exemplary model structure was presented for high strength and controlled release as a result of both ionic and photocrosslinking.

After targeting our 3D breast cancer model with OLE-mALG (200 µg/mL, 7 h), level of Bax apoptotic protein increased, while the level of Bcl-2 decreased (Fig. 5a). We considered the results of WST-1, western blot analyses (Fig. 5a and b), and the literature to determine the time and concentration range at which OLE activity can be observed in MCF-7 CSCs [6, 7, 30]. In addition, at the end of the viability assay with WST-1 analysis, it was determined that the cells remained alive at the rate of 70% after the OLE treatment. In this concentration and time interval, the apoptotic effect can be observed but the cells still maintain their viability. Similar to our results, in a study it was shown that OLE reduces cell viability, increases the level of reactive oxygen species, and inhibits cell migration and invasion in ER-negative breast cancer cells by modulating NF-kB pathway [31]. Another study presented that OLE induces p53-mediated apoptosis by up-regulating Bax and p53 genes and suppressing Bcl-2 in MCF-7 cells [32].

We evaluated the effect of OLE-mALG on tumorigenic properties in a 3D breast cancer model created with MCF-7 CSCs (Fig. 5b and c). As a result, we found that while E-cadherin protein level was increasing with OLE-mALG in cells, Slug and Vimentin decreased compared to control. Correlation of low E-cadherin and high Vimentin expression with distant metastasis has been reported by other researchers [6, 33]. In addition, Slug transcription factor is known suppresses the expression of E-cadherin, causing the cell to gain motility and plays a role in EMT [34]. Based on our findings, it can be said that OLE plays an EMT suppressive role in the breast cancer model. We also found an increase in all investigated gene expressions due to the OLE-mALG treatment, except CYCLIN D1 (Fig. 5c). p21 causes the cells to be kept in the S-phase and causes cell death in breast cancer cells by preventing proliferation in these cells [7]. In our study, OLE-mALG treatment resulted in increased expression of pluripotency and stemness genes CSCs (27-fold OCT3-4, 10-fold NANOG, 4-fold SOX2) in the 3D model of breast cancer. As a result of 2 mg/mL OLE application in glioblastoma CSCs, a decrease in OCT4 and SOX2 gene expressions, which are the target of miR-137, has been reported [10]. It was also stated in the literature that pluripotency markers play a role in the poor prognosis of cancer [35]. These studies were conducted with heterogeneous tumor population and since CSCs constitute a rare part of this population, the contribution of these genes to CSC tumorigenicity has not been well examined. Besides, it has been suggested that OCT4 and its pluripotency markers function differently in tumor-initiating CSCs than in embryonic stem cells [36].

The mechanisms underlying oleuropein’s anticancer effects on CSCs involve modulation of various signaling pathways implicated in CSC survival and proliferation, such as Wnt/β-catenin, Notch, Hedgehog, and PI3K/Akt/mTOR pathways. Moreover, oleuropein possesses potent antioxidant and anti-inflammatory properties, which may contribute to its anticancer activity by reducing oxidative stress and inflammation in the tumor microenvironment. Overall, oleuropein holds promise as a natural compound for targeting CSCs in cancer therapy. Further research is necessary to elucidate its precise mechanisms of action, optimize its delivery methods, and evaluate its efficacy and safety in clinical settings.

## Conclusions

In this study, we mimicked an in vivo system by creating a 3D model using MCF12A and HUVEC cells to represent chemotherapy/radiotherapy-resistant CSCs derived from MCF7 cells. The apoptotic effect of OLE was demonstrated separately in MCF7 and CSCs by showing a decrease in the anti-apoptotic Bcl-2 protein expression and an increase in the pro-apoptotic Bax, Caspase-3 and Caspase-9 levels. Then, OLE was loaded into mALG with a microfluidic based method for its controlled release. This is the first of its kind to use OLE-encapsulated microparticles by photocrosslinking with visible light using triethanolamine (TEA), N-vinylcaprolactam (VC) and Eosin Y disodium salt. We examined the effects of OLE on cell viability (WST-1), apoptosis (Bax, Bcl-2, caspase-3, caspase-9), EMT (Vimentin, Slug, E-kaderin), proliferation (CYCLIN D1, p21, SURVIVIN) and stemness (OCT3/4, NANOG, SOX2) and we concluded that this molecule plays a suppressive role in EMT, proliferation and tumorigenicity on CSCs. It was determined, through Western Blot analysis, that OLE-mALG suppressed the EMT transition of CSCs in the 3D model, as evidenced by decreased Vimentin (1.3-fold), Slug (2-fold), and increased E-cadherin (1.5-fold) protein levels. Furthermore, the expression of stemness genes, which are crucial for CSCs, such as OCT3/4 (increased by 27-fold), SOX2 (increased by 4-fold), and NANOG (increased by 10-fold), was found to be elevated in the 3D model after the application of mALG. Our results suggest that, mALG supports stemness characteristics while suppressing EMT in the 3D CSC model we established.

## Supplementary information

Below is the link to the electronic supplementary material.

ESM 1 (TIF 1.77 MB)

ESM 2 (TIF 3.81 MB)

ESM 3 (TIF 2.68 MB)

ESM 4 (TIF 4.31 MB)

ESM 5 (TIF 4.32 MB)

ESM 6 (TIF 237 KB)

ESM 7 (TIF 17.3 MB)

ESM 8 (DOCX 7.37 MB)

## Data Availability

The data that support the findings of this study are available on request from the corresponding author.

## Abbreviations

3D three: Dimensional
CSC: Cancer stem cell
DPBS: Dulbecco’s phosphate-buffered saline
EMT: Epithelial to mesenchymal transition
GelMA: Methacrylate gelatin
HT: Hydroxytyrosol
MA: Methacrylic anhydride
MACS: Magnetic-Activated Cell Sorting
Methocel: Methylcellulose
HUVEC: Human umbilical vein endothelial cells
mALG: Methacrylated alginate
MSC: Mesenchymal stem cell
OLE: Oleuropein
OLE-mALG: Oleuropein encapsulated in methacrylated alginate
PAGE: Polyacrylamide electrophoresis gel
TEA: Triethanolamine
VC: N-vinylcaprolactam
