# Supplementary figures and images for: Targeting resistant breast cancer stem cells in a three-dimensional culture model with oleuropein encapsulated in methacrylated alginate microparticles
Source: Daru. 2024 May 9;32(2):471–83. doi: 10.1007/s40199-024-00512-3 (PMC11555036; doi:10.1007/s40199-024-00512-3)

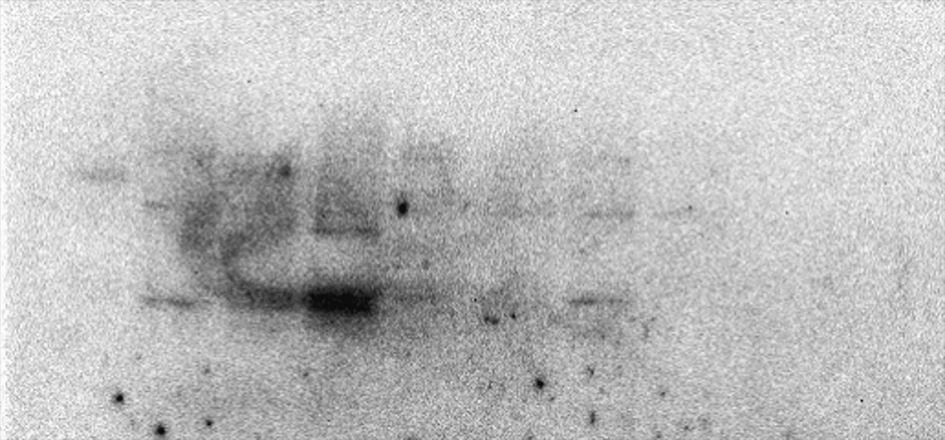

Supplement: Supplementary file 1 — (TIF 1.77 MB) [file 40199_2024_512_MOESM1_ESM.tif]

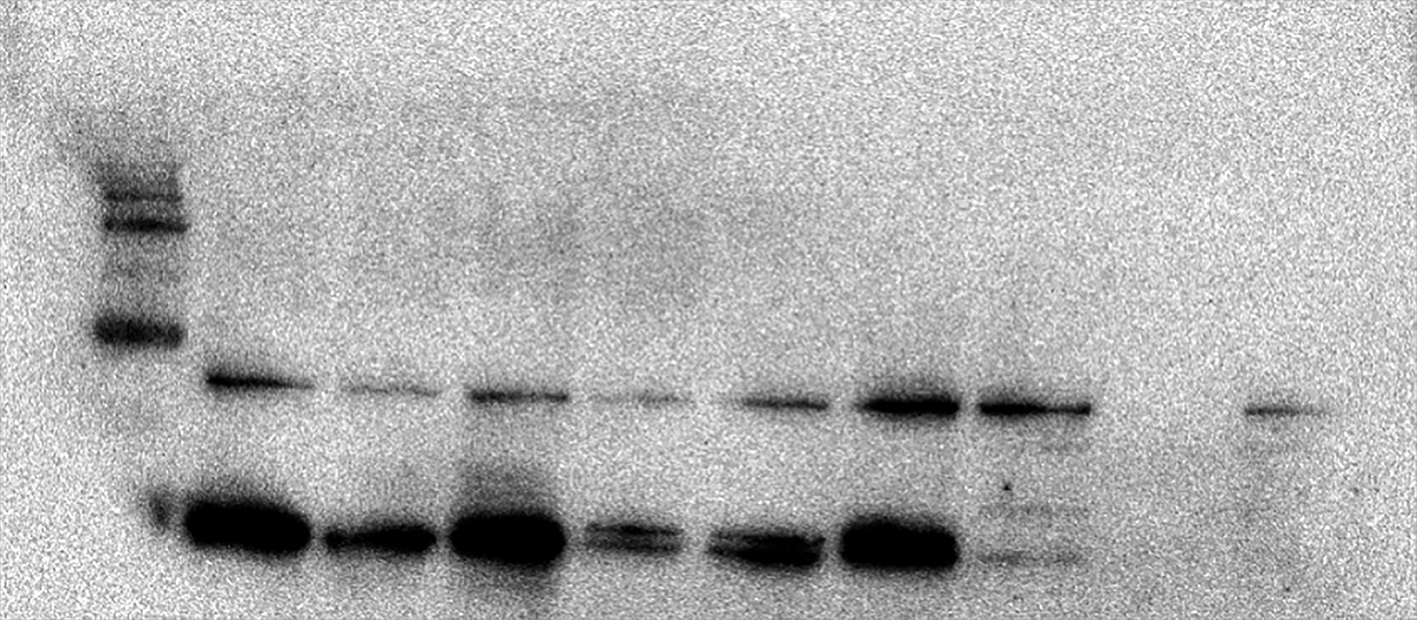

Supplement: Supplementary file 2 — (TIF 3.81 MB) [file 40199_2024_512_MOESM2_ESM.tif]

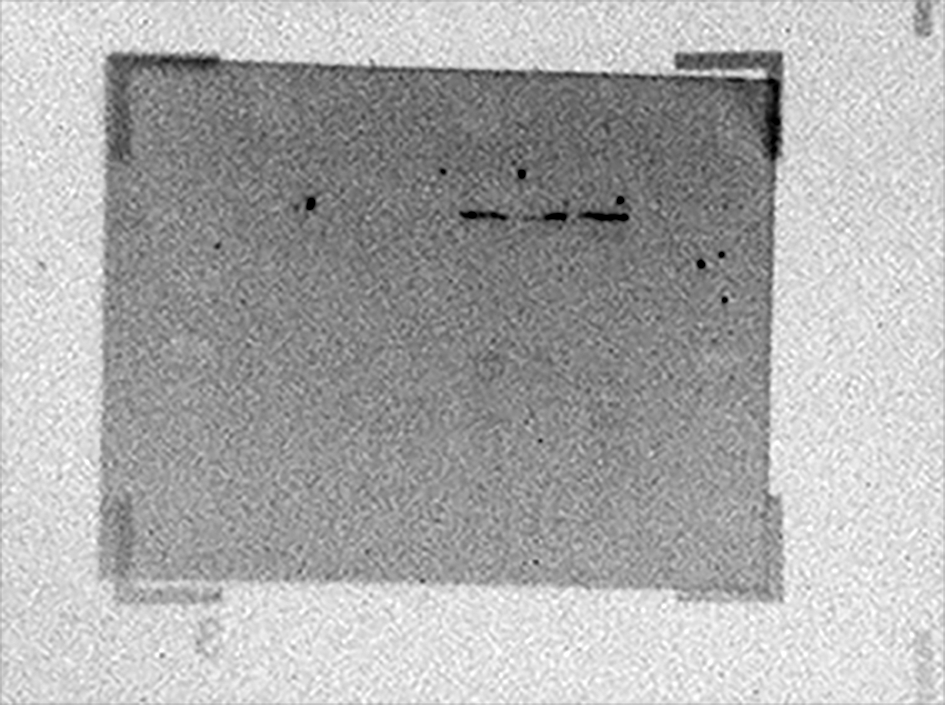

Supplement: Supplementary file 3 — (TIF 2.68 MB) [file 40199_2024_512_MOESM3_ESM.tif]

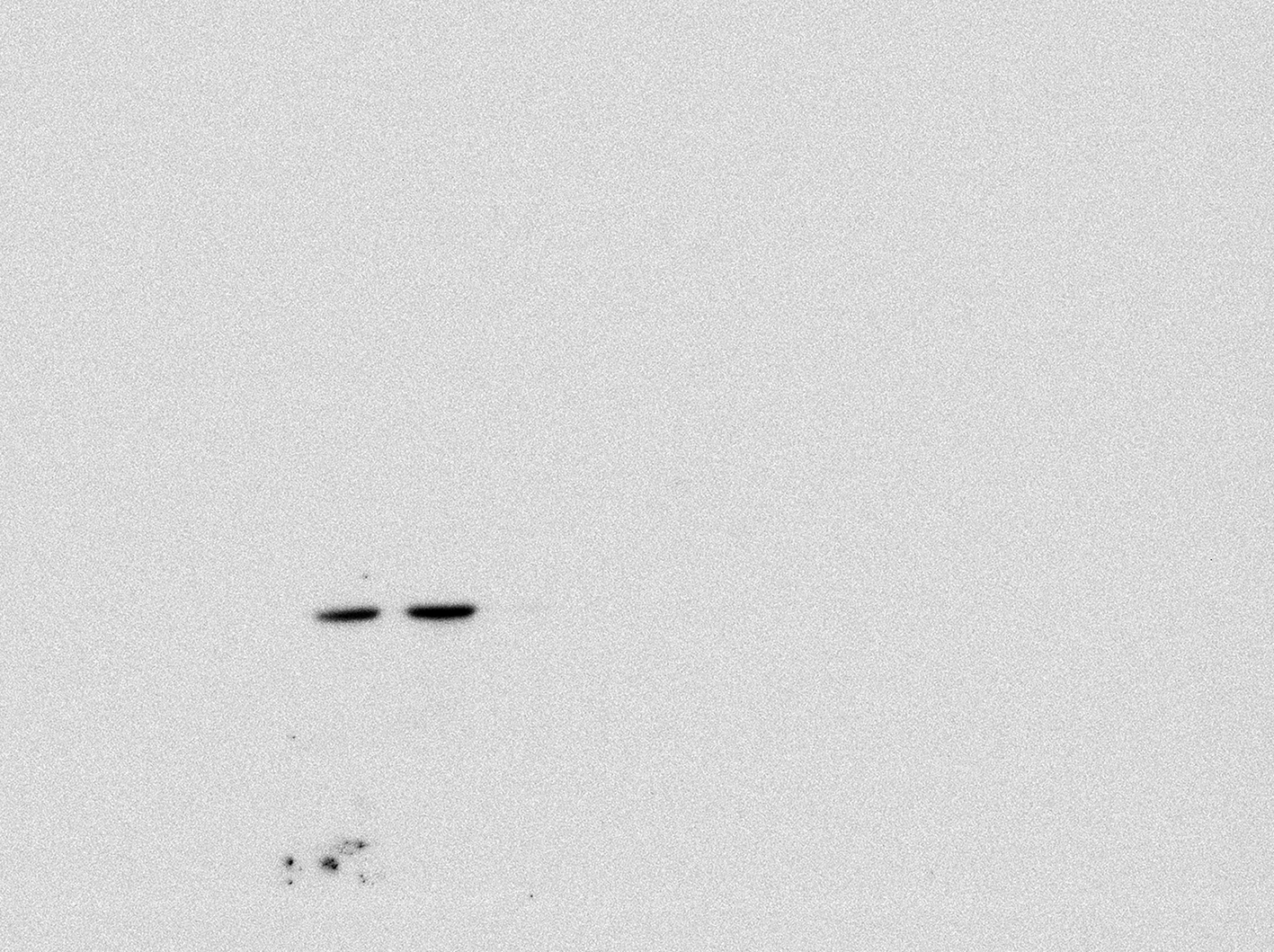

Supplement: Supplementary file 4 — (TIF 4.31 MB) [file 40199_2024_512_MOESM4_ESM.tif]

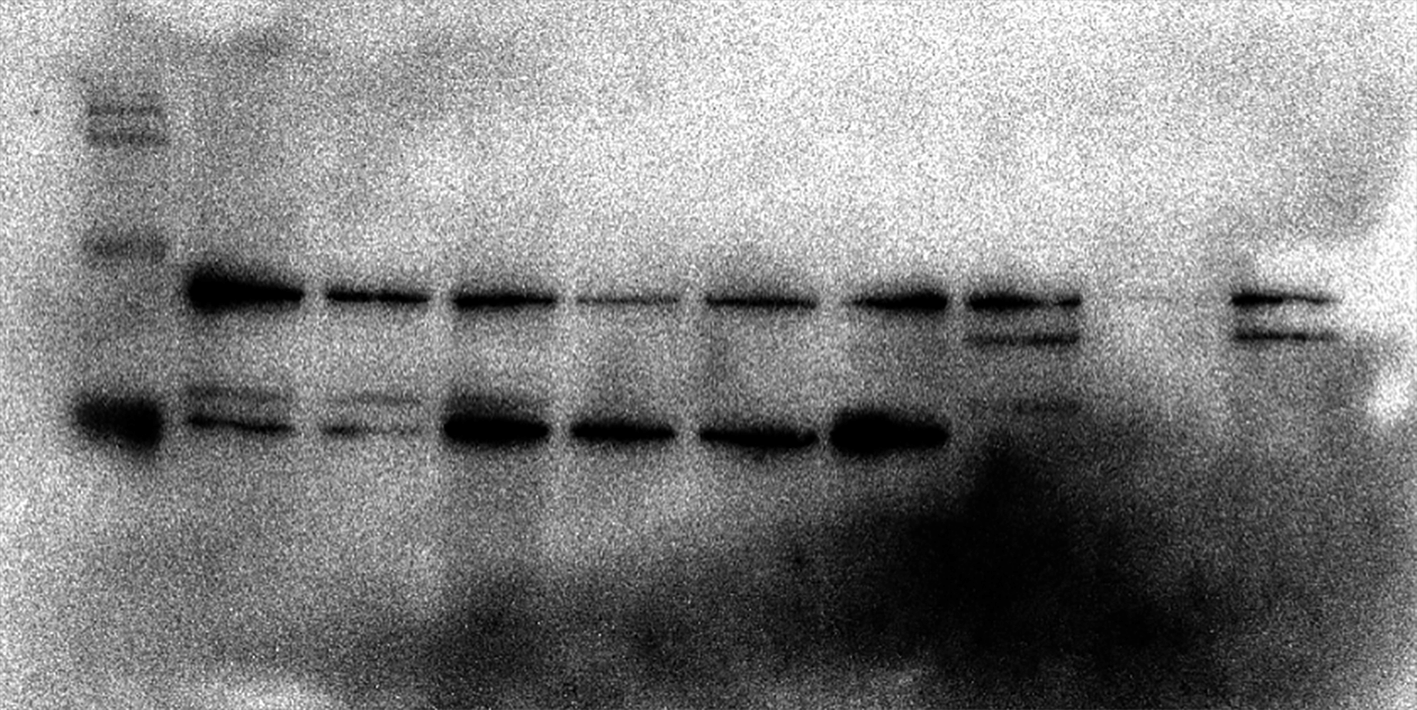

Supplement: Supplementary file 5 — (TIF 4.32 MB) [file 40199_2024_512_MOESM5_ESM.tif]

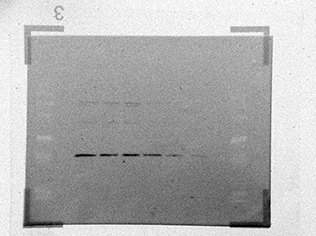

Supplement: Supplementary file 6 — (TIF 237 KB) [file 40199_2024_512_MOESM6_ESM.tif]

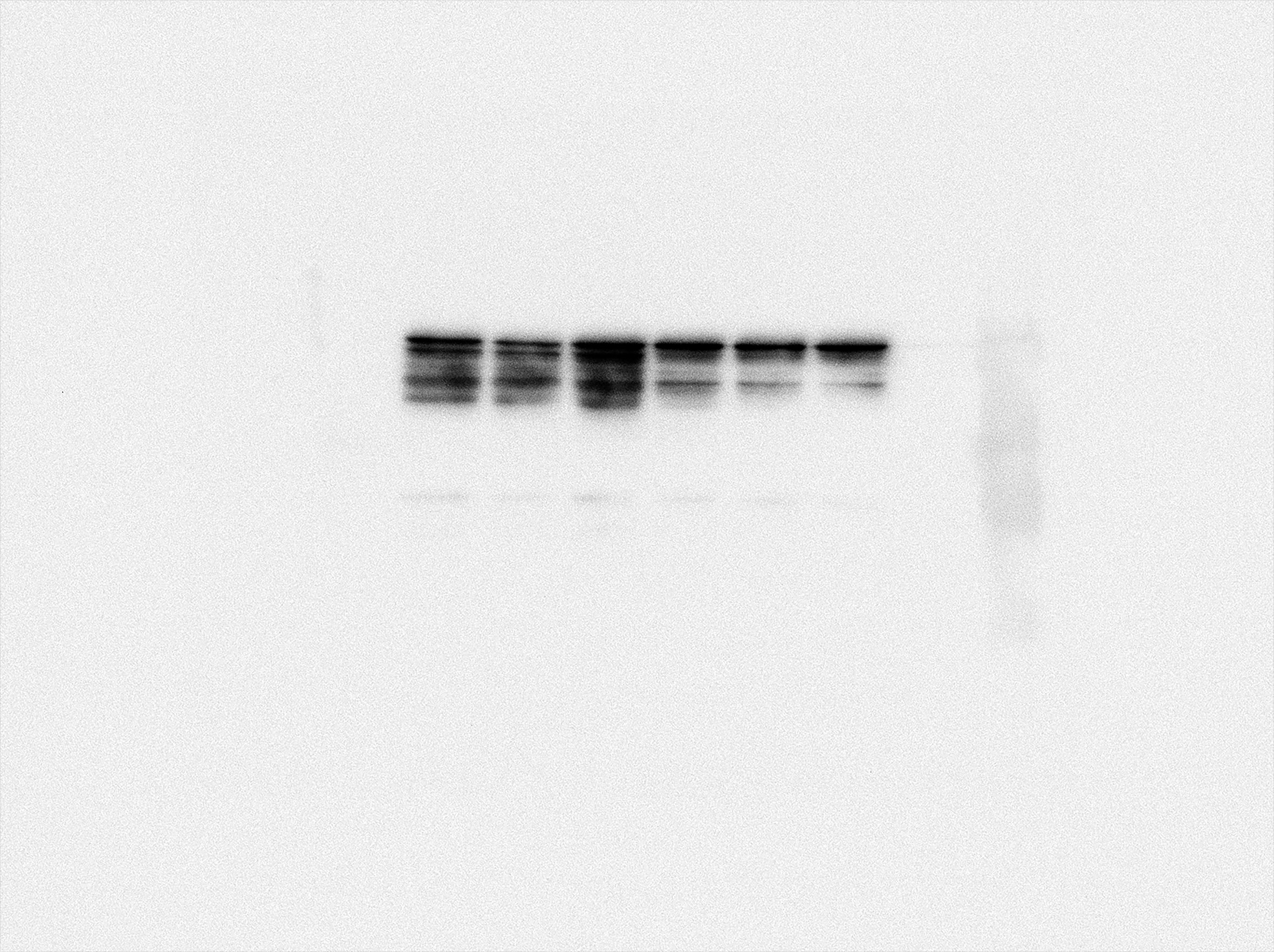

Supplement: Supplementary file 7 — (TIF 17.3 MB) [file 40199_2024_512_MOESM7_ESM.tif]
