# Supplementary material for: Targeting resistant breast cancer stem cells in a three-dimensional culture model with oleuropein encapsulated in methacrylated alginate microparticles
Source: Daru. 2024 May 9;32(2):471–83. doi: 10.1007/s40199-024-00512-3 (PMC11555036; doi:10.1007/s40199-024-00512-3)
Supplement: Supplementary file 8 — (DOCX 7.37 MB) [file 40199_2024_512_MOESM8_ESM.docx]

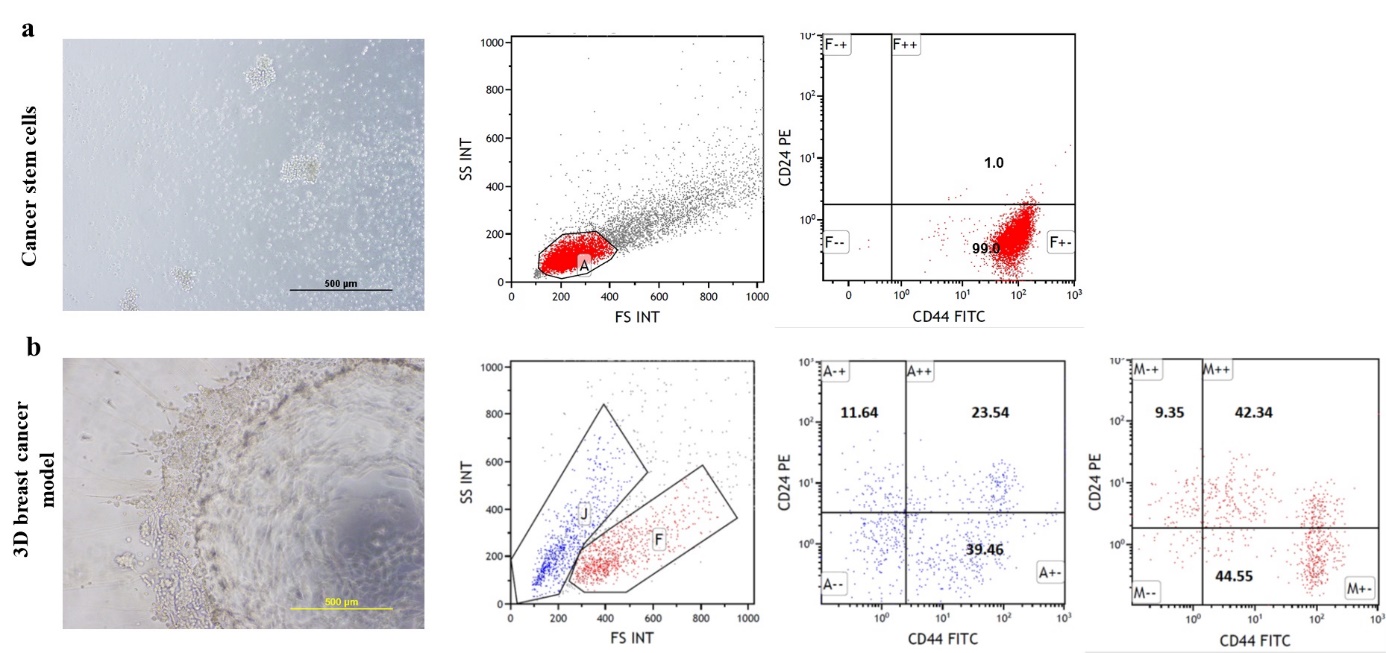


**Fig. Suppl. 1.** Characterization of the cancer stem cells and derived organoid model. a) Representative images and flow cytometric analyze of cancer stem cells (100% CD44+ and 1% CD24- phenotype). b) Representative images and flow cytometric analyze of cells co-cultured (inside the organoid) for seven days. Scale bar: 500 µm, 2.0 mm. The presence of two distinct populations with CD44 and CD24 characteristics within the created 3D structure was determined. The cells with CD44+/CD24+ phenotype within this two distinct populations are %23.54 and %42.34, respectively.

|  | **a** | **b** | **c** |
| --- | --- | --- | --- |
|  | **MCF7** | **CSC** | **MCF12A** |
| **Bcl-2** | 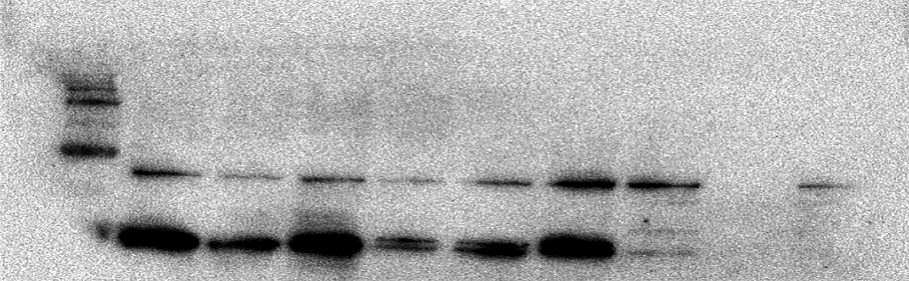 | 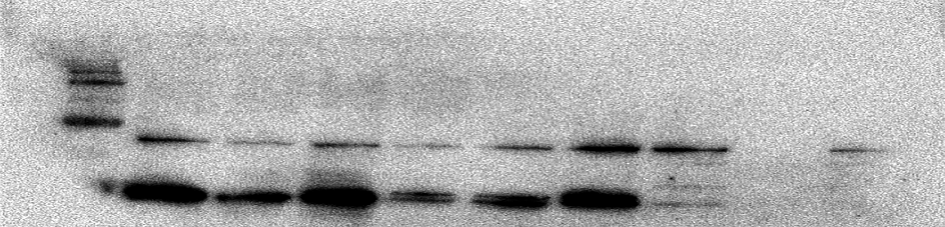 | 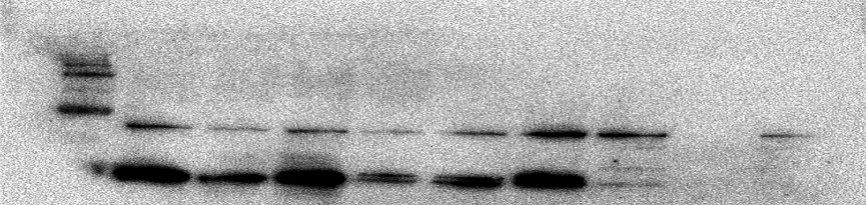 |
| **Bax** | 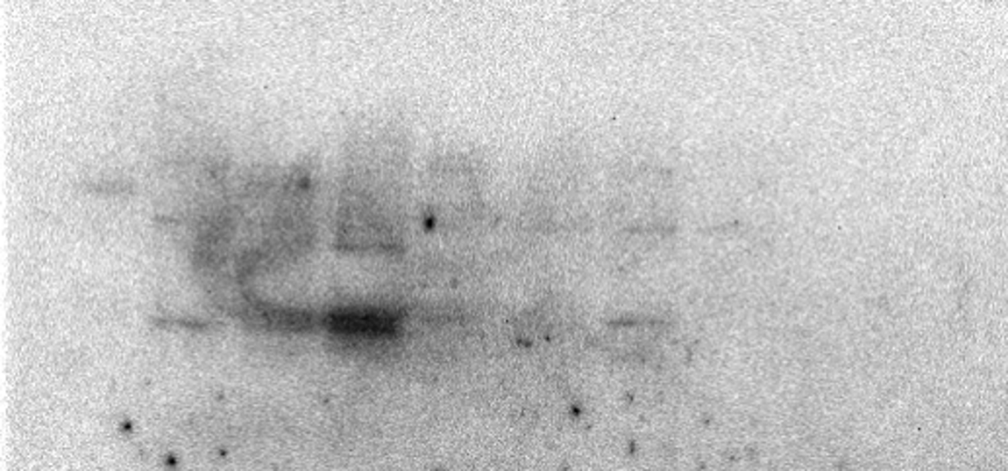 | 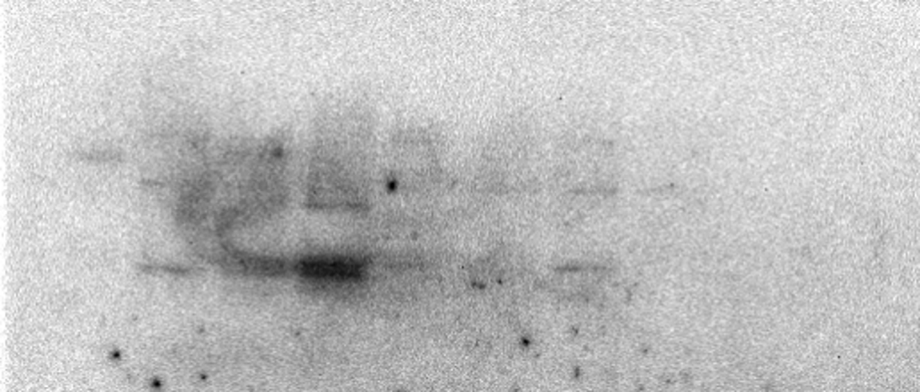 | 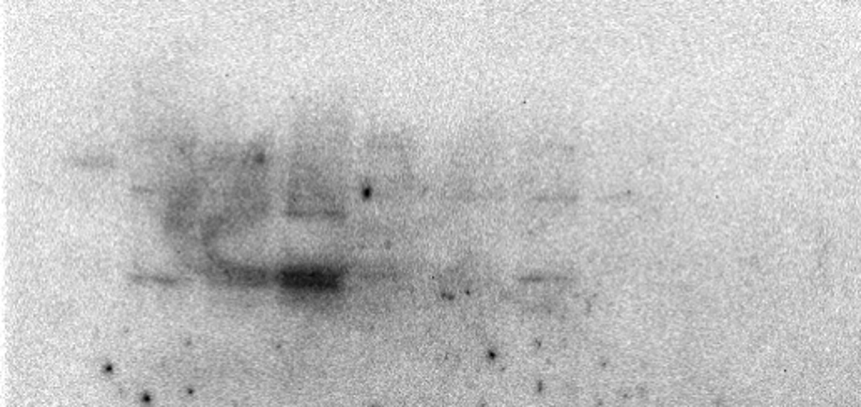 |
| **Caspase-3** | 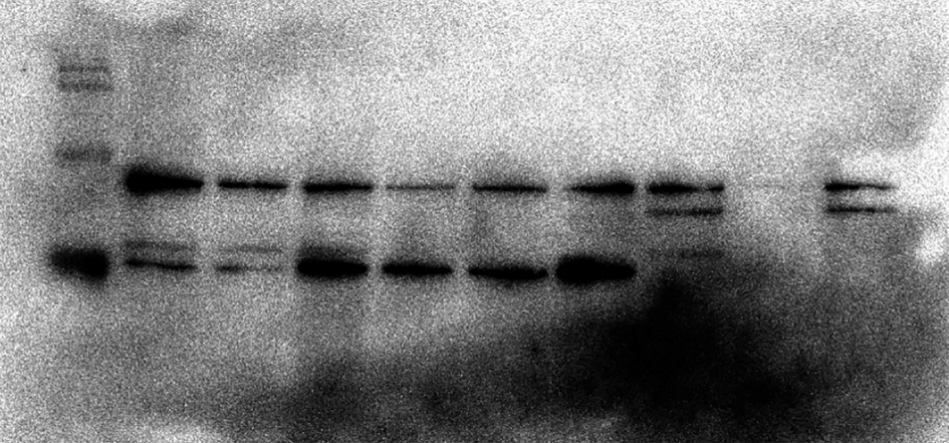 | 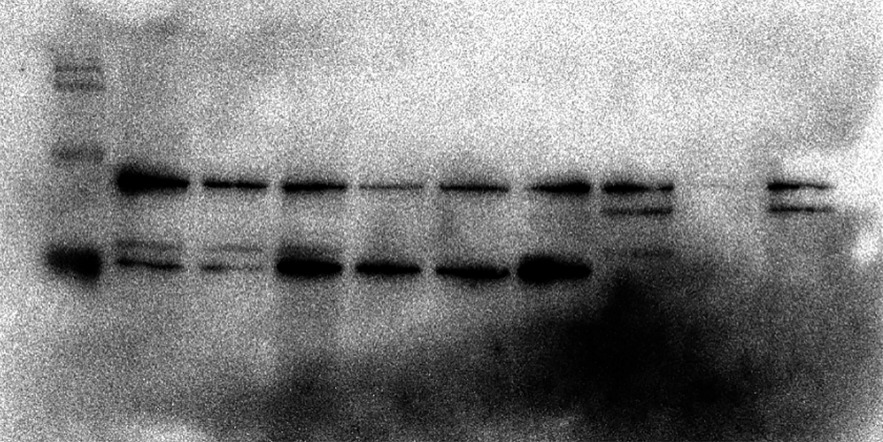 | 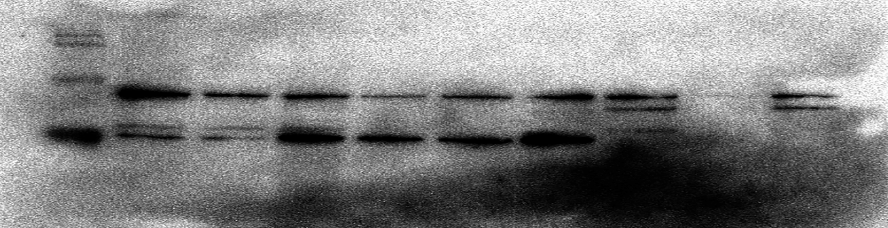 |
| **Caspase-9** | 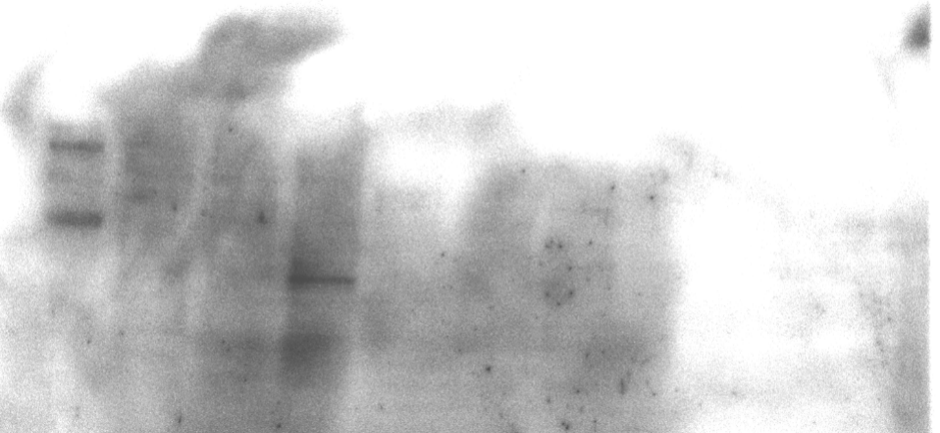 | 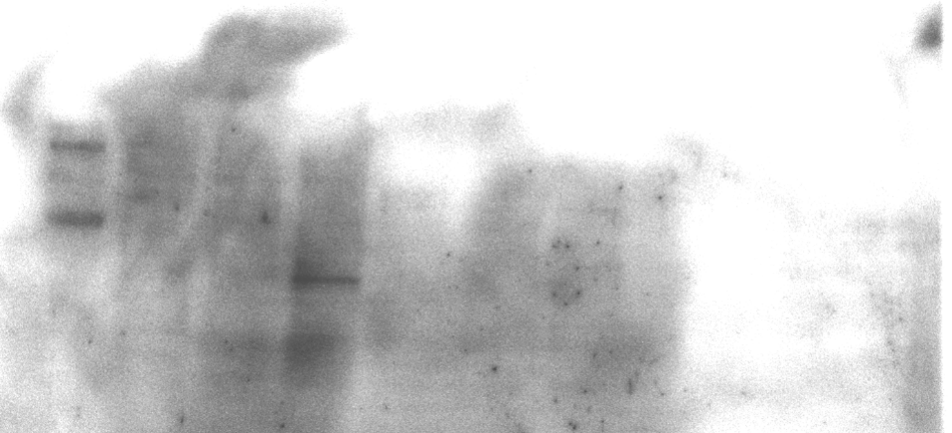 | 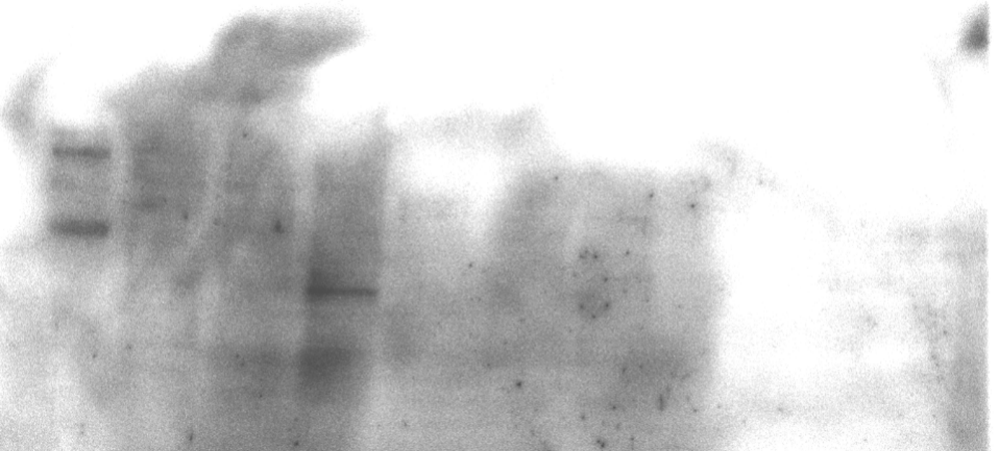 |
| **ß-actin** | 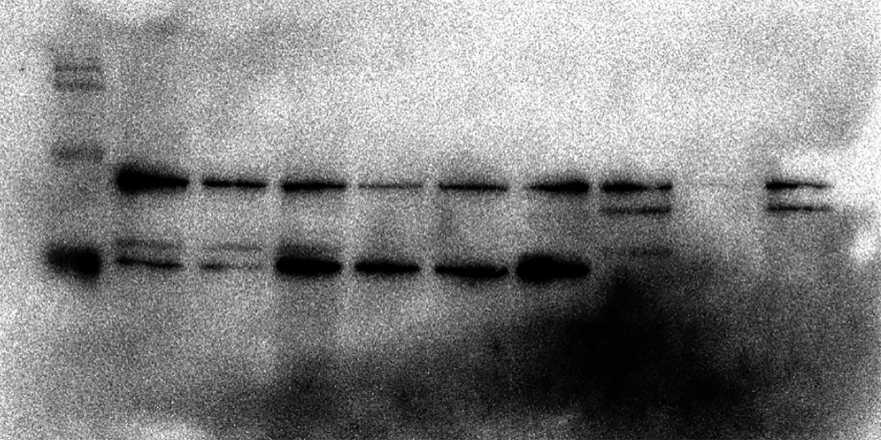 | 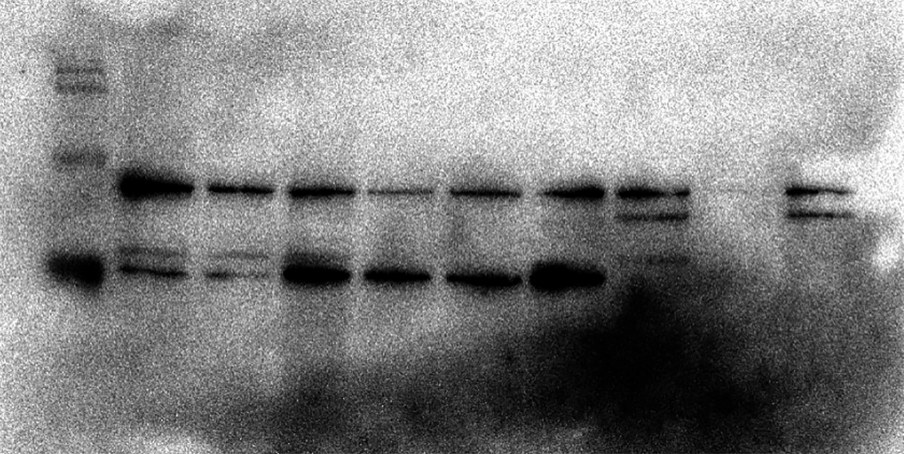 | 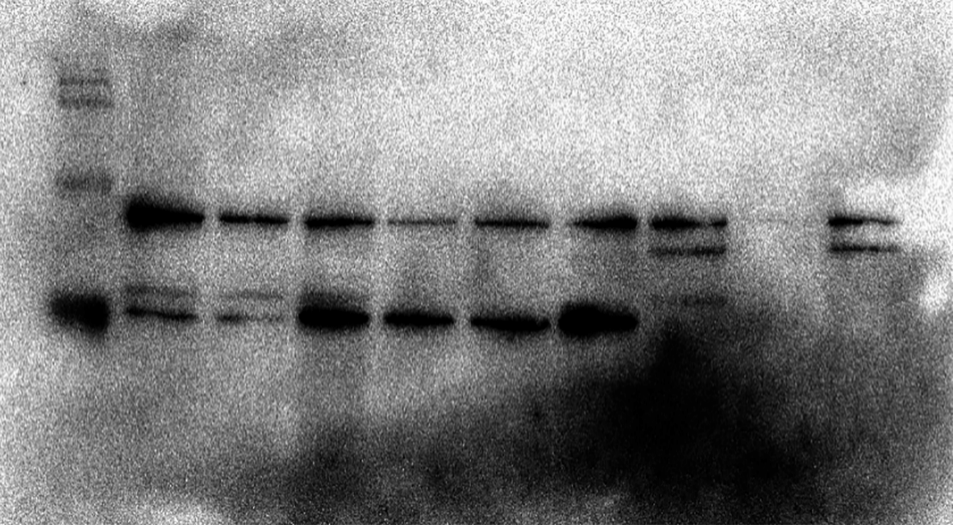 |
|  | **Control 4h 7h** | **Control 4h 7h** | **Control 4h 7h** |
|  | **Treatment time (hours)** | | |

**Fig. Suppl. 2.** Western blot images were obtained to examine apoptotic markers in MCF-7, MCF-7 derived CSCs, and MCF-12A cells before and after treatment with 200 µg/mL OLE for 0, 4, and 7 hours (7h). The results revealed an increase in apoptotic Caspase-3, Caspase-9, and Bax protein levels in MCF-7 cells after 7 hours of OLE treatment (see Figure Suppl. 2.A). A similar increase in Caspase-3 levels was observed in CSCs following the application of 200 μM OLE for 7h. Notably, there were no changes in apoptotic protein expressions detected in the anti-tumorigenic MCF-12A cells.
